# Supplementary figures and images for: Transcriptomic Analysis Reveals Cu/Zn SODs Acting as Hub Genes of SODs in Hylocereus undatus Induced by Trypsin during Storage
Source: Antioxidants (Basel). 2020 Feb 17;9(2):162. doi: 10.3390/antiox9020162 (PMC7070240; doi:10.3390/antiox9020162)

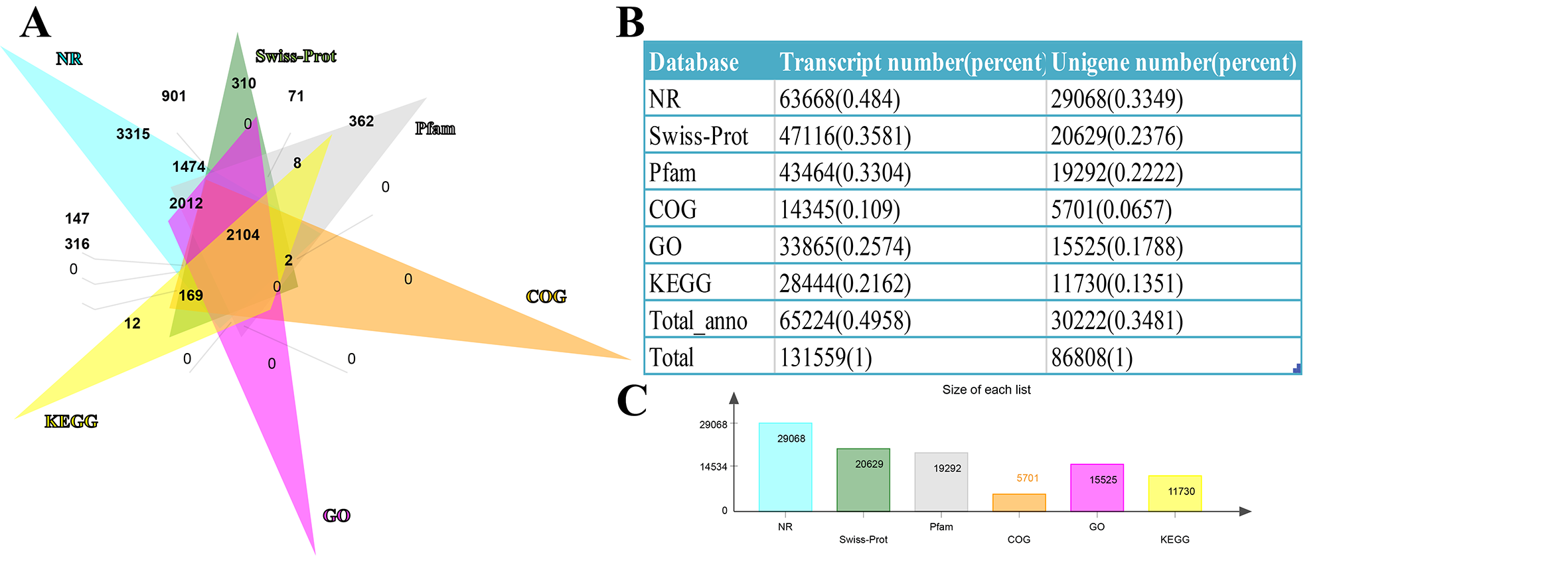

Supplement: Supplementary file 1 [file antioxidants-09-00162-s001.zip › antioxidants-699062-supplementary-revised-2/Fig. S1 Venn.tif]

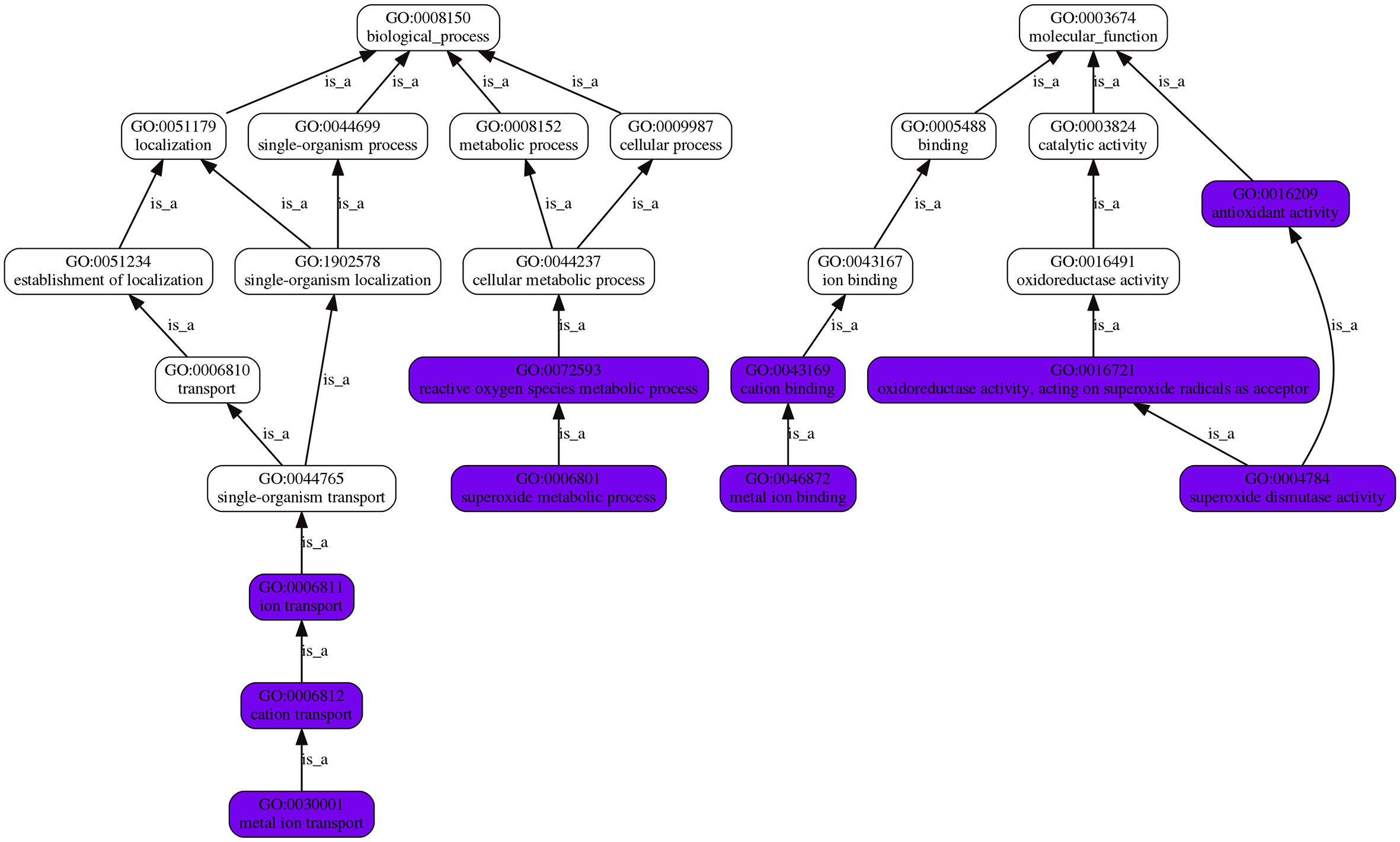

Supplement: Supplementary file 1 [file antioxidants-09-00162-s001.zip › antioxidants-699062-supplementary-revised-2/Fig. S10 DAG-SODup4.tif]

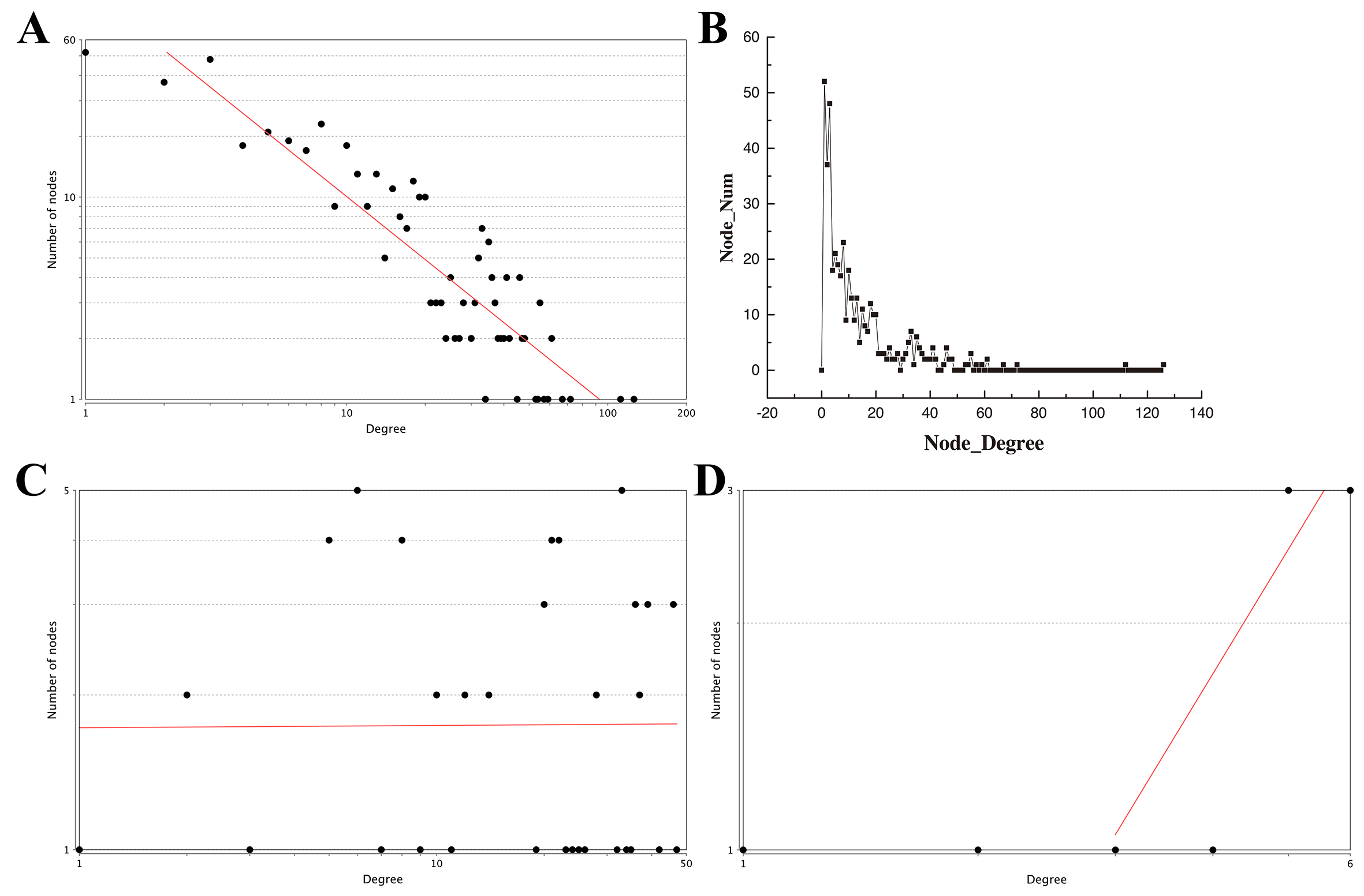

Supplement: Supplementary file 1 [file antioxidants-09-00162-s001.zip › antioxidants-699062-supplementary-revised-2/Fig. S13 Powerlaw.tif]
